# National responses to global health targets: exploring policy transfer in the context of the UNAIDS ‘90–90–90’ treatment targets in Ghana and Uganda

**DOI:** 10.1093/heapol/czx132

**Published:** 2017-10-11

**Authors:** Ellen McRobie, Fred Matovu, Aisha Nanyiti, Justice Nonvignon, Daniel Nana Yaw Abankwah, Kelsey K Case, Timothy B Hallett, Johanna Hanefeld, Lesong Conteh

**Affiliations:** 1 Department of Infectious Disease Epidemiology, School of Public Health, Imperial College London, London, UK; 2 School of Economics, Makerere University, Kampala, Uganda; 3 Policy Analysis & Development Research Institute, Kampala, Uganda; 4 Department of Health Policy, Planning & Management, School of Public Health, College of Health Sciences, University of Ghana, Accra, Ghana; 5 Department of Social and Behavioural Sciences, School of Public Health, University of Ghana, Accra, Ghana; 6 Anthropology, Politics and Policy Group, Department of Global Health and Development, Faculty of Public Health and Policy, London School of Hygiene and Tropical Medicine, London, UK; 7 Health Economics Group, School of Public Health, Imperial College London, London, UK; 8 Medical Research Council Centre for Outbreak Analysis and Modelling, Department of Infectious Disease Epidemiology, School of Public Health, Imperial College London, London, UK

**Keywords:** Policy analysis, HIV, UNAIDS, targets, networks

## Abstract

Global health organizations frequently set disease-specific targets with the goal of eliciting adoption at the national-level; consideration of the influence of target setting on national policies, programme and health budgets is of benefit to those setting targets and those intended to respond. In 2014, the Joint United Nations Programme on HIV/AIDS set ‘ambitious’ treatment targets for country adoption: 90% of HIV-positive persons should know their status; 90% of those on treatment; 90% of those achieving viral suppression. Using case studies from Ghana and Uganda, we explore how the target and its associated policy content have been adopted at the national level. That is whether adoption is in rhetoric only or supported by programme, policy or budgetary changes. We review 23 (14 from Ghana, 9 from Uganda) national policy, operational and strategic documents for the HIV response and assess commitments to ‘90–90–90’. In-person semi-structured interviews were conducted with purposively sampled key informants (17 in Ghana, 20 in Uganda) involved in programme-planning and resource allocation within HIV to gain insight into factors facilitating adoption of 90**–**90**–**90. Interviews were transcribed and analysed thematically, inductively and deductively, guided by pre-existing policy theories, including Dolowitz and Marsh’s policy transfer framework to describe features of the transfer and the Global Health Advocacy and Policy Project framework to explain observations. Regardless of notable resource constraints, transfer of the 90**–**90**–**90 targets was evident beyond rhetoric with substantial shifts in policy and programme activities. In both countries, there was evidence of attempts to minimize resource constraints by seeking programme efficiencies, prioritization of programme activities and devising domestic financing mechanisms; however, significant resource gaps persist. An effective health network, comprised of global and local actors, mediated the adoption and adaptation, facilitating a shift in the HIV programme from ‘business as usual’ to approaches targeting geographies and populations.

Key Messages

- Transfer of the 90–90–90 targets, and associated policy goals and content, was evident in both rhetoric and action in both countries despite substantial resource and capacity constraints potentially faced in both settings; transfer was mediated by a well-coordinated network of global and local actors with little to no notable opposition among the parties engaged.
- There are a number of domestic financing plans in the pipeline to support progression to the 90–90–90 targets, but whether these can be established successfully and finances realized in the timeframe are unknown.
- Both countries continue to be in favour of ambitious health targets to help galvanize action and leadership, however, informants at the country level felt strongly that recognition for progress should be equally valued to target attainment given variability in baseline performance and that there should be greater support for countries that fall behind.

## Introduction

The establishment of global health targets, intended for adoption at the national level, seeks to focus the attention of funders, implementers and national-level policy-makers. Setting of targets or goals has occurred in global health since the late 1950s with the global Smallpox Eradication Programme, but has become increasingly commonplace, spanning multiple health issues, as evidenced by the Millennium Development Goals and subsequent Sustainable Development Goals (SDGs) (United Nations; Shiffman and Smith 2007). The HIV response alone has had numerous, arguably ambitious, targets: ‘3 by 5’ to treat three million people living with HIV (PLHIV) by 2005; ‘15 by 15’ to treat 15 million PLHIV by 2015; ‘getting to zero’ for zero new HIV infections, zero AIDS-related deaths and zero discrimination by 2015 (World Health Organization 2003; UNAIDS 2011, 2015). In 2014, the Joint United Nations Programme on HIV/AIDS (UNAIDS) set the ‘90–90–90’ targets that operationalize what countries need to do, and what programme coverage targets need to be attained at certain time points, to achieve the policy goal of ending the AIDS epidemic by 2030, as advocated for in the SDGs (UNAIDS 2014; Stover *et al.* 2016) (Box 1). The 90–90–90 targets differ from previous targets, such as 3 by 5; rather than being set arbitrarily, mathematical modelling has accompanied the 90–90–90 targets, utilized to derive the programme coverage levels and estimate the resource needs for achieving the policy goal of ending the AIDS epidemic by 2030 (Stover *et al.* 2016). Similar strategies have been developed using modelling for the epidemics of malaria and tuberculosis (TB) (Stop TB Partnership 2015; World Health Organization 2015).

**Box 1.** Summary of UNAIDS’ 90–90–90 strategy: policy goal, targets and associated policy content

**The ‘90**–**90**–**90’ treatment targets set by UNAIDS**	
**Policy goal**	To eliminate the AIDS epidemic by 2030.
**Policy target**	90% of all PLHIV to know their status, 90% of those with diagnosed HIV infection to receive sustained antiretroviral therapy (81% of PLHIV), and 90% of those on antiretroviral therapy to achieve viral suppression (i.e. 73% of PLHIV) by 2020. Each target rises to 95% by 2030 to achieve the end of the AIDS epidemic.
**Policy content**	**First 90:** More frequent testing/increase demand for testing; strategic, focused testing to key population groups and/or geographies; increase/make available a broader number of HIV testing and counselling approaches, e.g. self-testing, provider initiated counselling and testing and community-based approaches. **Second 90:** Recommend antiretroviral therapy to all people with diagnosed HIV infection (or ‘test and treat’), without the requirement of a prior CD4 test; use of preferred, optimized regimens; make HIV treatment and care (including diagnostic tests and other treatment-related items) free to the individual; address implementation issues that have often slowed scale-up, e.g. frequent drug stockouts, barriers to procurement of optimally affordable medicines and diagnostics, and inadequate availability of second- and third-line regimens. **Third 90:** Sustained use of HIV treatment and ongoing virologic monitoring to verify treatment success and to intervene to support treatment adherence and re-engage those who fall out of care; every person starting HIV treatment will need to have access to viral load testing.

**Box 2.** Details of the theoretical frameworks applied

**Theoretical frameworks drawn upon to measure the extent of transfer and consider factors contributing to observations in country case studies**	
**Dolowitz and Marsh** (**1996**) **framework**	**Global health advocacy and policy project framework**
Comprised of six questions (A–F) to help examine all aspects of the policy transfer process	Three categories comprised of 10 factors that variously could influence effectiveness of global health networks in attracting attention, resources, and influencing policy outcomes.
Applied to enable examination of policy transfer process in both settings and thus compare differences between settings	Applied to gain insight into which factors facilitate transfer and explain observed differences between settings

A. What is transferred and how is transfer demonstrated? B. What are the degrees of transfer? C. What restricts or facilitates transfer? D. From where are lessons drawn? E. Who are key actors involved in the transfer process? F. Why do actors engage in transfer?	Network and actor features: factors internal to the network (1) leadership, (2) governance, (3) composition, (4) framing strategies utilized Policy environment: factors external to the network (5) allies and opponents, (6) funding availability, (7) norms. Issue characteristics: features of the problem the network seeks to address (8) severity, (9) tractability, (10) affected groups.

Whilst HIV programmes in many sub-Saharan African countries are well established, and have achieved tremendous scale up in access to antiretroviral treatment (ART) in the past decade, the 90–90–90 targets have been met with scepticism, and even criticism, for being over ambitious (Katabira and Oelrichs 2007; El-Sadr *et al.* 2012; Sidibé *et al.* 2016). The utilization of mathematical modelling for informing programme decisions across a range of countries, such as the analysis accompanying the 90–90–90 targets and the policy goal to end AIDS necessitates generalization of intervention costs and impact. Most mathematical modelling studies, such as this, do not capture the full range of budget and health system constraints that may limit the feasibility of approaches deemed cost-effective from such analyses (Mikkelsen *et al.* 2017). Constraints include resource (human and financial) and health system capacity, ease with which such resources can be reallocated and not based on historical patterns, costs of implementing change and demand for services (Hauck *et al.* 2016; Mikkelsen *et al.* 2017). Complex public health interventions, such as those advocated for in 90–90–90, present a wide range of context specific constraints that may prohibit ease of adoption that must be considered by decision-makers alongside the benefits of responding to such targets.

In sub-Saharan Africa, where the burden of HIV is greatest, many health facilities are overburdened and under-resourced and initiating such high proportions of the population of PLHIV into care has the potential to exacerbate pre-existing weaknesses in care provision, such as stock-outs of essential commodities and human resource challenges (Roberts *et al.* 2016; Rosen and Fox 2011). For the targets to be met, a substantial shift is needed from the strategy of broad nationwide programme scale up, to more targeted and active promotion and provision of testing, treatment and care that is cognizant of the geographic and demographic heterogeneity of the epidemic. An estimated 36 billion USD is required globally per year between 2015 and 2020 to fund implementation; however, in 2013 an estimated 19 million USD was mobilized for the HIV response, which falls far short of this new requirement (UNAIDS 2014). Thus, efforts to prioritize programmes and seek efficiencies will also need to be supplemented by domestic resource mobilization given that donor financing for the HIV response has been stabilizing or declining in recent years (Vassall *et al.* 2013; Remme *et al.* 2016). National level decision-makers are faced with a complex decision problem regarding how best to target limited resources in order to achieve expanded coverage of treatment and care services.

Debates on the value of global targets or vertical programmes in low-income countries and their impact on health systems have prevailed for many years. Arguments against include concerns that targets can obfuscate efforts to achieve universal health care coverage by channelling resources toward one health concern that may not have the greatest effect on population health or be aligned with priorities of the government (De Maeseneer *et al.* 2008; Dieleman and Haakenstad 2015). In low-income settings where resources are highly limited, the potential opportunity cost of reallocation of health resources to meet just one disease specific target may be great. An alternative view, however, is that some targets are adopted in rhetoric only, as a vision to guide the response, but to a large extent do not influence programme activities or resource allocation and thus have minimal impact on health. Here targets are of little concern, but are ultimately ineffective at achieving policy change. Evaluation of the Expanded Programme on Immunization for the eradication of polio found that, while successful in galvanizing attention, action and strengthening immunization programmes more broadly, in some settings there was diversion of resources to meet this health goal at the expense of other health issues or the wider development agenda (Sutter and Cochi 1997; Taylor *et al.* 1997; Loevinsohn *et al.* 2002). Such findings raise concerns against global target setting, thus we consider it of value to examine the process by which targets are adopted and whether they influence programme planning and resource allocation.

In this research, we aim to explore what the influence of UNAIDS’ 90–90–90 targets have been on the national HIV programme in Ghana and Uganda. We assess how these technical and ambitious targets have been transferred, or adopted and adapted, to the national level and whether the transfer of the targets appears in rhetoric only, or if notable policy, programme and budgetary changes have occurred to support implementation (we do not assess implementation). We utilize an integrated analytical approach that combines the policy transfer framework developed by Dolowitz and Marsh to consider what has been transferred how this is evidenced, and constraints on transfer, and supplement this with Global Health Advocacy and Policy Project (GHAPP) conceptual framework for the effectiveness of global health networks to provide deeper reflection on the role of actor networks in mediating the observed response to the 90–90–90 targets (Dolowitz and Marsh 1996; Shiffman *et al.* 2015). We draw on multiple sources of information, documents of donors and national policy-makers and key informant interviews, to triangulate findings and minimise bias.

## Methods

### Setting

Case studies of the response to 90–90–90 were conducted between August 2016 (25 months following UNAIDS’ release of 90–90–90) and April 2017 in Ghana and Uganda. The countries were selected purposively for their differences in current HIV burden, historic response to the epidemic, and economic status (Table 1). Uganda has high HIV burden and a long history of engaging different actors to implement a variety of approaches to combat the epidemic; how the low-income nation responds to these ambitious targets in this context is thus of interest (Allen *and* Heald 2004; Parkhurst *et al.* 2002). Ghana has a low HIV prevalence relative to Uganda but also exhibits lower treatment coverage. Whilst Ghana has experienced economic growth to reach lower-middle income status this necessitates greater counterpart financing for the HIV programme; whether the country prioritises resources to respond to these targets is thus of interest (Ghana AIDS Commission 2015). Selection was also somewhat opportunistic given pre-existing relationships with senior policy-makers that would enable access to the relevant informants for the study.

**Table 1.** HIV programme and funding indicators in Ghana and Uganda (UNAIDS 2016; UNAIDS 2016; Institute of Health Metrics and Evaluation 2016; Granich *et al.* 2016; The World Bank 2016).

	Ghana	Uganda
**HIV epidemic indicators**		
Estimated HIV prevalence, 15–49, Spectrum (year of estimate)	1.6% (1.3–1.9%) (2015)	7.1% (6.6–7.7%) (2015)
Estimated number of people living with HIV, Spectrum (year of estimate)	270 000 (230 000–330 000) (2015)	1 400 000 (1 300 000–1 500 000) (2015)
**90**–**90**–**90programmeindicators**		
Estimated percentage of PLHIV aware of their status (year of estimate)	40%^a^ (2014)	69% (2015)
Estimated percentage of diagnosed on treatment (year of estimate)	30% of PLHIV^a^ (2014)	81% (60% of PLHIV) (2015)
Estimated percentage on treatment that are virally suppressed (year of estimate)	Unknown nationally^a^ (2014)	Unknown nationally^b^ (2015)
**Funding indicators**		
Total HIV spending in US$ (year)	81 677 333 (2011)	579 700 000 (2013)
HIV spending as % GDP (year)	0.21 (2011)	3.91 (2009)
Primary funders of the HIV programme	The Global Fund	PEPFAR (>70% of HIV/AIDS programme)

a Limitations in reporting systems result in uncertainty in ascertaining real numbers tested, in care, and retained. Estimates from modelling are uncertain.

b One study showed 90% viral suppression, but sample was only 42% of estimated population on ART.

### Document analysis

Documents relevant to the national HIV response in Ghana and Uganda were searched for by the primary researcher from websites of the National AIDS Commission (NAC), Ministry of Health (MOH), President’s Emergency Plan for AIDS Relief (PEPFAR), The Global Fund and Country Coordinating Mechanism (the two major funders of the HIV programme in both countries) from July 2014 onwards (the date UNAIDS released 90–90–90—at the International AIDS Society conference in Melbourne). Key informants were also asked to share documents released post July 2014 that may have included national HIV policies, programme or budget information. Twenty-three documents were obtained (14 Ghana, 9 for Uganda). The document review allowed for tracing of transfer over time. Documents were assessed for presence or absence of alignment to the 90–90–90 targets in rhetoric (defined as statements regarding the adoption or alignment to the targets or whether the country is ‘on track’ to meet the targets), actual or planned policy or programme changes, and actual or planned budgetary changes (such as seeking programme efficiencies, donor or domestic resource mobilization or re-allocation). Details on policy and programme changes were extracted and compared against recommendations from UNAIDS’ 90–90–90 strategy to assess whether a shift in approach from ‘business as usual’ had occurred. A second reviewer verified data extraction.

### Key informant interviews and analysis

In each country, semi-structured interviews were conducted with purposively sampled individuals in the MOH, NACs, AIDS Control Program, organizations providing programme funding or technical assistance [Department for International Development; The Global Fund; World Health Organization (WHO); UNAIDS; United Nations’ Children’s Fund (UNICEF); US Government entities such as PEPFAR, Centers for Disease Control and Prevention (CDC), and United States Agency for International Development (USAID)], and major programme implementers. Informants were selected first by sensitizing each key organization and asking senior staff to nominate individuals for interview based on their role in the response to 90–90–90 and knowledge of programme, policy or budget changes. Informants were asked for names of other key individuals from the same or other organizations crucial in the response until representatives from all major organizations cited by respondents as important in the response were covered from both countries; thus resulting in a slightly different profile of those interviewed based on the composition of each network responding to 90–90–90 in each country. Interviews were conducted in-person and individually, except for three interviews in Ghana (two conducted by telephone and one with three informants). Thirty-seven interviews were conducted with key informants, lasting on average 60 min (ranging from 40 to 105 min) (Table 2). All interviews were conducted in English. Interviews were also held with informants in malaria and TB programmes to enable comparison of the response to other diseases and assess perceptions of financing for HIV from individuals working outside HIV. Interviews were audio recorded where consent was given (two respondents, one from Ghana and one from Uganda, asked to not be audio recorded) and transcribed. Transcripts were managed and analysed thematically in Dedoose. Coding was initially deductive applying the pre-existing policy frameworks of Dolowitz and Marsh and GHAPP, with additional inductive coding cycles. Qualitative interview data was triangulated with the document review. Results are presented where informants contradicted findings from the document review. Emerging themes and summary findings from each country were reviewed by local study teams and discussed. Findings were shared with key informants in both countries to assess if findings were a fair reflection of the response in their country; enabling further validation of the findings.

**Table 2.** Interviews conducted in Ghana and Uganda

Profession	Ghana	Uganda
Ministry of Health or National Health Service	1	2
Ghana/Uganda AIDS Commission	3	4
Programme implementers	3	4
Global actors providing technical assistance and/or funding	7	3
External consultants	2	1
Other (academics, civil society organizations)	0	3
Individuals involved in the response to malaria or TB	1	3
**Total**	**17**	**20**

### Theoretical framework

Policy transfer theory explores how ‘knowledge about policies, administrative arrangements, institutions and ideas in one political setting (past or present) are used in the development of policies, administrative arrangements, institutions and ideas in another political setting’ (Dolowitz and Marsh 1996). National responses to global health issues appear increasingly guided by ideas put forward by organizations in the international community; in particular, in low- and middle-income settings where financial and technical assistance are often provided in support of adoption of policies or acceptance of norms. We utilize elements of two pre-existing theoretical frameworks to uncover the policy transfer process that has occurred in Ghana and Uganda. Dolowitz and Marsh’s (1996) framework poses six questions to examine the policy transfer process (Box 2). The framework encourages evaluation of the extent to which transfer has occurred and is evident, in addition to what potential constraints on transfer exists that may prohibit transfer, both of which are of value to our investigation into the manner in which the 90–90–90 targets have been adopted in the current environment of financial uncertainty and health system capacity constraints (Box 2) (Dolowitz and Marsh 1996). We integrate this framework with the GHAPP framework to consider the involvement of actor networks at local and global levels influencing the response, as opposed to vertical or horizontal analyses commonly applied in policy transfer studies. Furthermore, while Dolowitz and Marsh consider the rationale for transfer on a spectrum—from voluntary to more directly coerced—the network features, policy environment and issue characteristics considered in the GHAPP framework are used to consider why such a pattern of transfer was observed. This allows a more nuanced comparison between the two case study countries. Thus, questions D to F of Dolowitz and Marsh are expanded upon utilizing aspects of the GHAPP framework (Box 2).

### Ethical approval

Ethical approval was obtained from the authors institutes in the UK, Ghana and Uganda.

## Results

In presenting our results we first respond to questions A–C of the Dolowitz and Marsh framework where we assimilate findings from key informants and the document review. Considerations about how the transfer was mediated, to help explain similarities and differences, in both settings are later discussed utilizing the GHAPP framework in reference to questions D–F of the Dolowitz and Marsh framework. Findings are summarized in Table 3.

**Table 3.** Summary of policy transfer in both settings utilizing Dolowitz and Marsh (1996) policy transfer framework

	(A) What is transferred & what is the evidence of transfer?	(B) To what degree has the policy been transferred?	(C) What are the constraints on transfer?	(D) From where are lessons drawn?	(E) Who are key actors involved in the transfer process?	(F) Why do actors engage in transfer?
**Summary findings***(presented as common to both settings or specific to either country)*	**Both:** The 90–90–90 treatment targets (also the policy goal of eliminating the AIDS epidemic, and much of the associated policy content) and concepts for more targeted programming that is cognizant of geography and population groups. In addition the need to increase programme efficiency and mobilize resources domestically. This was evidenced in rhetoric with all key informants in both settings and in policy, programme and budgetary documents (but to a lesser extent for the latter). **Ghana:** Latest NSP oriented entirely around 90–90–90 targets, meeting the targets is a core aim, is mentioned upfront in chairman's letter indicating high level commitment, and has separate sections are devoted to each 90 target stating which interventions will be implemented to meet that target. However, indication from informants that less is ready for implementation other than roll out of test and treat as supported by PEPFAR and The Global Fund. **Uganda:** More limited mention of 90–90–90 in national strategic and operational documents, but very clear, targeted approach in PEPFAR Country Operational Plans.	**Both:** Whilst many documents demonstrate a direct copy of the target, upon further investigation across operational and implementation documents, a more modest set of targets appear to have been adopted or are to be aimed for. **Ghana:** The focus is predominantly on the first two 90s as access to viral load machines is limited. For the third 90 there is an emphasis more on health systems strengthening and adherence initiatives. **Uganda:** National strategic plan and PEPFAR Country Operational Plans contain targets for second 90 of 80% opposed to 81%. It is not clear why this slightly lower target has been adopted—one informant said it was as a consequence of a modelling analysis of what programme coverage levels could realistically be achieved with anticipated available resources.	**Both:** Inadequate financial resources available for commodities for the response: HIV test kits, antiretroviral treatment for treating all PLHIV, and viral load testing. Health system capacity constraints, such as human resources to support higher patient loads. Complex policy targets that may require application of mathematical modelling to guide programme decisions, but capacity at the local level for such approaches is relatively limited. Large-scale programmatic shift required from approach of broad nationwide scale up to targeted approach, including shift in the manner in which resources are allocated for the response. **Ghana:** Uncertainty in epidemiological information at lower administrative levels results in difficulty in prioritizing resources lower than the regional level. Resources are being allocated to improve data quality and for monitoring of progress toward 90–90–90. Previously lack of political support for targeted approaches as did not want to appear not equitable in service provision. **Uganda:** Appears to be greater flexibility in reallocation of resources to more targeted approach as PEPFAR are the major funder of the HIV programme and thus have more autonomy with which to decide how to reallocate compared with government resources. Therefore, fewer constraints on transfer in Uganda.	**Both:** From international organizations and implementing agencies. Notably directly from UNAIDS’ 90–90–90 strategy document, derived from mathematical modelling study. Decision-makers in both countries reported using in-country experience about effective interventions. Lessons learned from new modelling studies in both countries in addition to the application of epidemiological information to consider which geographies and populations to prioritize in the response.	**Both:** Well-coordinated health network present in both settings, clearly defined roles, comprised of actors at local and global levels: National/local: NAC, MOH, major local implementers Global: WHO, UNAIDS, and US Government entities (CDC, PEPFAR, USAID), The Global Fund (but to a lesser extent in both countries in the first two years after 90–90–90 was released as both countries were in the middle of an existing funding period) **Ghana:** Private companies (in particular, oil industry) through corporate social responsibility agreements, resource mobilization officer (UNDP funded) EQUIP providing technical assistance in implementation for responding to 90–90–90 Health Policy Plus providing modelling for scenarios for the National Strategic Plan **Uganda:** Civil society organizations, e.g. NAFOPHANU (hosted the 90–90–90 country launch) Futures Institute—modelled and costed scenarios for 90–90–90 for consideration Irish Aid To a lesser extent than in Ghana—private sector, groups such as the Uganda Olympics Committee	**Both:** Whilst the reasons for adoption of the targets are many there are substantial constraints operating the may limit the rationality of implementing these targets, in particular when considering the cost required in terms of overall benefit to population health as a decision maker. There is a clear amount of international pressure to respond to the targets – in many cases countries may have signed on to respond to the targets before even conducting studies into the feasibility of implementation. Respondents in both settings cited the feeling of international pressure to adopt the targets regardless of available funding. Summary: Transfer of 90–90–90 is voluntary as was not a pre-requisite for receipt of funding, driven by perceived necessity as a mandate to the UN and for international acceptance/conforming to global norms to respond. However, to receive funding from PEPFAR for ‘test and treat’ both countries needed to revise their treatment guidelines—for this specific policy this could therefore be argued as more a direct imposition. **Ghana:** 90–90–90 transfer utilized as an advocacy tool to secure additional funds due to counterpart funding needs.

### (A) What has been transferred and how is transfer demonstrated?

All respondents across both settings stated the national HIV response in their country had adopted the 90–90–90 targets with the majority stating the targets had influenced significant policy, programme and budgetary changes. This was largely supported by findings from the document review; Table 4 presents an overview of the national documents reviewed. Documents from both settings evidence adoption of the 90–90–90 targets in rhetoric, in addition to policy, programme and budget changes intended to support implementation to meet the targets. In both countries, adoption of the 90–90–90 targets, and planned programme and budgetary support for 90–90–90 is first witnessed in PEPFAR’s Country Operational Plans and is recognized in national strategic (August 2016 in Uganda and September 2016 in Ghana), policy and operational documents subsequently. Ten (71%) documents in Ghana and 4 (44%) documents in Uganda detail proposed, planned or actual programme or policy changes to respond to 90–90–90. Of interest, many key informants in Uganda commented on the alignment of the latest National Strategic Plan (NSP) to 90–90–90; however, the targets were only mentioned once on page 64 (compared with 55 mentions in the Ghanaian NSP). Two respondents said this was because the targets came late in the development of the NSP, whereas more respondents (*n* = 6) said 90–90–90 came at the perfect time for inclusion in the strategy. Although not attributed to 90–90–90, the programme targets in the Ugandan NSP are closely aligned to the global targets—the target treatment coverage for PLHIV is 80% by 2020 (also stated as 80% in the 2015 PEPFAR Country Operational Plan, but referred to as ‘national saturation’) as opposed to the 81% of the 90–90–90 targets.

**Table 4.** Review of documents for the national HIV response in Ghana and Uganda to assess influence of UNAIDS’ 90–90–90 targets

No.	Document title (author)	Date document published	Document type	Number of mentions/ number of pages	Rhetoric	Policy or programme changes	Budgetary changes
**Ghana**							
1	The costs and impact of investing in the HIV response in Ghana (Health Policy Project, USAID, PEPFAR)	April 2015	Strategic	8/42	–	X	X
					Pre-adoption of 90–90–90 by Ghana—modelling to consider feasibility of adopting targets^a^	Proposed/scenario analysis for 90–90–90	Modelled estimates of resource needs for 90–90–90 Total cost of 450 million for NSP scenario including attainment of 90–90–90
2	Ghana Country Operational Plan 2015 (PEPFAR)	August 2015	Operational	22/74	X	X	X
					Adopted 90–90–90 for key populations The PEPFAR Ghana targets are to achieve 90–90–90 among 60% of key populations in in high burden regions/districts in southern Ghana by the end of 2017. Not on track to meet 90–90–90 for whole population, only sufficient ART for 50% of PLHIV	Core plans for 90–90–90 for key populations onlyPredominantly first 90 (HIV counselling and testing for key populations in 5 scale up regions and in Cape Coast) but support for lab services for testing and viral load monitoring and retention with ‘Models of Hope’ programme	For key populations only Resources allocated for programme activities (for example, lab, HIV counselling and testing)
3	Making strategic choices: Prioritizing HIV interventions in a resource limited setting. Options for Ghana's next National Strategic Plan (USAID, PEPFAR, Health Policy Plus)	September 2015	Strategic	19/59	–	X	X
					Pre-adoption of 90–90–90 by Ghana—modelling to consider feasibility finds: ‘Even if such significant resource mobilization was successful, unless the country addresses the health systems and policy gaps identified in this study, it will not achieve its ultimate goal of getting 90 percent of PLHIVs to know their status, 90 percent of them to be on treatment, and 90 percent of those on treatment to be virally suppressed’^a^	Proposed/scenario analysis for 90–90–90 Need to focus on indirect (reducing stigma, health systems strengthening) as well as direct interventions otherwise will not achieve 90–90–90	Modelled estimates of resource needs for 90–90–90 80 million USD for treatment alone pa and 2015 NSP budget for treatment was US$19 million—therefore massive resource scale up required
4	Ghana Country Operational Plan 2016 (PEPFAR)	June 2016	Operational	22/70	X	X	X
					Does not specifically say that Ghana has adopted the targets	Plans for achieving first and second 90, with systems strengthening to assist in preparation for meeting third 90	Allocations for some activities relating to 90–90–90 but not all
5	90–90–90 Roadmap to Treat All (National AIDS Control Program)	July 2016	Operational, presentation	4	X	X	–
					Adopted 90–90–90 and Fast Track strategy	Plans for second 90	
6	Locate, test, treat, and retain (L2TR) 90–90–90 Ghana Campaign (National AIDS Control Program)	July 2016	Operational	61/20	X	X	–
					Adopted 90–90–90 and Fast Track strategy	Plans for 90–90–90, in particular the ‘one million community health workers project’	
7	Guidelines for antiretroviral therapy in Ghana (Ministry of Health, NACP, Ghana Health Service)	September 2016	Policy/guideline	2/134	X	–	–
					Adopted 90–90–90		
8	National HIV/AIDS Strategic Plan 2016–20 (Ghana AIDS Commission)	September 2016	Strategic	55/131	X	X	X
					Adopted 90–90–90 and Fast Track strategy	Plans for achieving first and second 90, with systems strengthening to assist in preparation for meeting third 90	
9	Ghana AIDS Commission launches the national HIV and AIDS strategic plan & ‘treat all’ policy (Ghana AIDS Commission)	2016	Press release	1/2	X	–	–
					Informed by 90–90–90 and inline with SDGs		
10	Treat All Implementation Update (NACP)	October 2016	Operational— presentation	2/29	–	X	X
						Actual activities for second 90	Need for reprogramming/reallocation of funds for 90–90–90
11	Guidance on the CCM Approach to develop the funding request 2018–20 (The Global Fund)	October 2016	Funding request	1/12	–	–	X
							NACP to consider modelling from USAID/Health Policy Plus for resource needs for 90–90–90
12	HIV/TB Funding Request 2018–20 (Ghana CCM)	December 2016	Funding request	10/33	X	X	X
					Adopted 90–90–90	Plans for first 90 and actual for second 90 Want to focus on PMTCT and early infant diagnosis as main way to increase diagnosis and coverage of ART	Planned and actual re-allocation/re-programming of TGF budget allocation, efforts to seek efficiency gains and prioritize programme activities to geography and populations in need, donor and domestic resource mobilization
						Plans for three 90s for key populations	
13	Prioritized above allocation request (Ghana CCM)	December 2016	Funding request	2/5	–	X	X
						To support planned and already ongoing activities	Funding request for Models of Hope and viral load monitoring
14	Ghana Global Fund 2017–19 Allocation Letter (The Global Fund)	December 2016	Funding letter	0/12	–	–	–
					Does state need to strengthen the cascade to meet the Fast Track targets but no mention of 90–90–90 specifically^a^		
**Uganda**							
1	Uganda Country Operational Plan 2015 (PEPFAR)	September 2015	Operational	7/104	X	X	X
					Adopted and on track ‘15 districts have already met the first two 90s’	Plans to focus on first two 90s for scale up districts and plans for nationwide scale up of VL	‘PEPFAR Uganda is able to make these shifts through an additional $30 million for treatment scale-up, further streamlining of the Country Operational Plan 12 core package of services, transitioning out of low burden/low yield districts and sites, rationalization of implementing partners, and exploration of more efficient service delivery models’.
2	National HIV/AIDS Strategic Plan 2015/16–19/20 (Uganda AIDS Commission)	August 2016	Strategic	1/87	X	–	–
					Strategy is ‘cognizant of the global and national commitment to end AIDS by 2030’ and aligned to the Fast Track Strategy and 90–90–90	Programme activities are not directly attributed to attainment of 90–90–90 goals in the NSP^a^	Estimated cost of implementing NSP goals is 3647.9 billion USD for 5-year period^a^
3	National HIV/AIDS Monitoring and Evaluation Plan 2015/16–19/20 (Uganda AIDS Commission)	August 2016	Operational	0/113	–	–	–
					Aligned to ‘end of AIDS by 2030’ but no mention of 90–90–90 specifically^a^		
4	National HIV/AIDS Indicator Handbook 2015/16–19/20 (Uganda AIDS Commission)	August 2016	Operational	0/97	–	–	–
5	National HIV/AIDS Priority Action Plan (Uganda AIDS Commission)	August 2016	Strategic	0/72	–	–	–
6	2015/16 Country Progress Report (Uganda AIDS Commission)	November 2016	Evaluation	25/82	X	X	–
					Adopted and on track	Actual changes for first year and planned up until 2020	
7	Consolidated HIV Prevention and Treatment Guidelines (Ministry of Health Uganda)	December 2016	Guideline	7/152	X	X	–
					Adopted 90–90–90	Actual policy change for all three 90s—treat all irrespective of CD4 cell count, differentiated service delivery, improved retention strategies	
8	Uganda Country Operational Plan 2016 (PEPFAR)	January 2017	Operational	32/65	X	X	X
					Adopted, and on track pending implementation of test and treat and new service delivery models to reduce issues with commodities for rapid scale up	Plans and actual ongoing activities for 90–90–90	Plans for efficiency gains through new service delivery models and prioritization of testing, treatment and care services by geography and location
9	National Multi-Sectorial HIV/AIDS Resource Mobilization Strategy 2015/16–19/20 (Uganda AIDS Commission)	April 2017	Strategic	3/89	X	–	X
					States national response is aligned to 90–90–90		Implementation of the strategy aims to raise 69 million USD toward the 3647 million USD needed for the NSP.90–90–90 is cited as a reason for increasing mobilization efforts, but the amount from these efforts targeted to meeting 90–90–90 is unclear.

a Information detailed is considered relevant although does not necessarily provide evidence of the transfer of 90–90–90.

**Key:**

**Number of mentions**:

One count given to a mention of ‘90–90–90’ and each of the sub goals, for example, ′first 90′ or the equivalent percentage for each category, i.e. 90%, 81% or 73%. Count only once per sentence, or a figure, and only once in table unless 90–90–90 is the content of cells in a table, in which case count each mention.

**Standardized data extraction form used for reviewing each document**:

1. **Rhetoric:** Statement of the nation’s adoption, commitment or alignment to the 90–90–90 treatment targets or Fast Track strategy, including statements about the country being ′on track′ to any or all three of the targets. Including problem statements and magnitude of the problem.

2. **Policy orprogrammechange:** Statements about 90–90–90 are supported by recommended, planned or implemented policy or program change intended to achieve 90–90–90 or each of its sub-goals.

3. **Budgetary changes:** Statements about 90–90–90 that are supported by recommended, planned or actual reallocation of resources, resource mobilization, and efforts to seek efficiencies (includes modelled estimates of resource needs and resource allocation).

Details on how the countries intend to respond to 90–90–90 as demonstrated in the documents reviewed and discussed in interviews are presented in Table 5 against recommendations from UNAIDS. Both countries demonstrate a move from broad nationwide scale-up to an approach that targets populations and geographies of highest burden to achieve optimal impact with the available resources, which demonstrates a substantial shift in approach for both countries. Arguably, Uganda demonstrates a more marked shift, rapidly moving to district programming, whereas Ghana first adopts a regional plan and focuses on prior gaps in nationwide intervention coverage, such as prevention of mother to child transmission. The following excerpt from PEPFAR Uganda’s Country Operational Plan summarizes the change toward programme prioritization by geography and population:

**Table 5.** Alignment of national HIV programme activities with recommendations by UNAIDS in 90–90–90 strategy document

UNAIDS 90–90–90 Strategy: policy content for each target	National-level adoption of 90–90–90: policy and programme activities planned and actual	
	Ghana	Uganda
**First 90: 90% of PLHIV to know their status**		
**More frequent testing/increase demand for testing.**	Nationwide ‘Know Your HIV Status’ campaign with special focus on high yield geographic locations and population groups. HIV-related stigma and discrimination reduction campaign. Provide HIV testing services at multiple service delivery points—health facilities including clinics, community and outreach services delivery points, special events etc. Expand cadre of service providers for outreach testing services.	Nationwide testing campaigns. Numerous campaigns for adolescents: peer-to-peer engagement, social media campaigns.
**Strategic, focused testing to key population groups and/or geographies.**	Increase testing in four regions with antenatal clinic prevalence >2%. Improve targeted testing of key populations starting with the four priority regions (Ashanti, Eastern, Greater Accra and Western) and Brong Ahafo (facility-based and outreach). Pregnant women and TB patients Outreach testing in the general population will not be done because of low yield but will continue for key populations.	Discontinue low-yield HIV testing service activities and concentrate on high-yield activities. 27 PEPFAR focus testing districts with regular monitoring to assess yield. High yield areas, e.g. fishing communities where HIV prevalence ranges between 14.9% and 35% around the shores of Lake Victoria, other lake systems throughout the country, and on the border with the Democratic Republic of the Congo. Deliver HIV testing services in male dominated work settings and areas where male access to services is poor. Test and start will minimize number of repeat tests required.
**Make available a broader number of HIV testing and counselling approaches, e.g. self-testing, provider initiated counselling and testing and community-based approaches.**	Reactivate provider-initiated testing services (PITC) to include testing of children on admission, emergency room testing, increased testing in DOTS corners, blood donor testing and diagnostic testing to include all Hepatitis B and C positive clients. Not doing nationwide PITC^a^	To improve access and efficiency of HIV testing services a mix of health facility and community-based approaches to be utilized. Differentiated models of HIV testing services have been developed: Facility based approaches: routine PITC that is ‘opt out’ and client initiated testing and counselling. Community based approaches: Index client testing—HBCT or snowball, outreach HIV testing services in hotspots and workplaces. Pilot studies for HIV self-test are underway and pending attainment of supporting evidence, guidelines on roll out will be developed by MOH. Increase partner notification strategies.
**Second 90: 90% of those diagnosed to be on treatment**		
**Recommend antiretroviral therapy to all people with diagnosed HIV infection, without the requirement of a prior CD4 test.**	Yes, but in a more staggered way as nationwide scale up of test and treat too expensive. ‘Test and treat’ rolled out in four regions 1 October 2016: Fast track enrolment of clients including key populations receiving clinical care Intensify current enrolment of HIV positive pregnant women, children, TB clients, hepatitis B and C clients, key populations and serodiscordant clients. Scale-up ART in TB DOTS sites and PMTCT sites From January 2017 implement ‘test and treat’ to remaining 6 other regions.	The Government of Uganda provided commitment to begin ‘test and treat’ in October 2016 pending reforms of the supply chain – to be conducted by PEPFAR and the MOH. ‘Test and treat’ was rolled out January 2017.
**Use of preferred, optimized regimens.**	Preferred first line regimens: tenofovir+emtricitabine or tenofovir+lamivudine+an NNRTI such as efavirenz.	Recommended first line regimen in accordance with WHO recommendations: tenofovir+lamivudine+efavirenz. All HIV-infected adults and adolescents aged 10 years and above should be initiated on tenofovir + lamivudine and efavirenz as a once-daily fixed dose combination.
**Make HIV treatment and care, including diagnostic tests and other treatment-related items, free to the individual.**	Not discussed in interviews or identified in documents reviewed.	Not discussed in interviews and not identified in documents, but HIV testing and treatment is thought to be free to the individual in Uganda.
**Address implementation issues that have often slowed scale-up, e.g. frequent drug stock outs, barriers to procurement of optimally affordable medicines and diagnostics, and inadequate availability of second- and third-line regimens.**	Reviews being undertaken. Consideration given to how to manage stock outs and high demand for treatment. In the event of deep and prolonged periods of ART stock outs, priority will be given to PLHIV with CD4 of less than 500.	Reviews being undertaken to streamline pipeline. Differentiated service delivery being implemented. Guidelines for reallocation of commodities between facilities and improvements in communication developed.
**Third 90: 90% of those on treatment to achieve viral suppression**		
**Sustained use of HIV treatment and ongoing virologic monitoring to verify treatment success and to intervene to support treatment adherence and re-engage those who fall out of care.**	Ghana's focus for the first half of the 90–90–90 time period is to focus on the first two 90s and then gradually draw focus on scale up of viral load. More efforts instead on adherence of patients on ART.	PEPFAR will support MOH’s transition to viral load testing for ART monitoring.
**Every person starting HIV treatment will need to have access to viral load testing.**	Current utilization of viral load machines considered to be < 10%. NACP in collaboration with PEPFAR and CDC will review laboratory policy and strategic plan, develop guidelines and scale up plans for viral load testing and train all laboratory staff on these documents. These guidelines would support an increase in the uptake of viral load. Implementation of the plan is scheduled to begin in the third quarter of 2017 supported by reprogrammed funds from The Global Fund (if awarded). The plan is to scale this up in 2018–20.	A plan was devised to increase the viral load tests from 100 000 in 2014 to 1.2 million tests per year in 2018.

a One informant reported routine PITC was not to be rolled out nationally due to limited resources.

*PEPFAR Uganda previously focused on national programmatic scale up with little emphasis on geographical prevalence, disease burden, or evidence of KPs [key populations]/PPs [priority populations]. During 2015 COP [Country Operational Plan] preparation, attention was paid to aligning district budgets and associated targets to disease burden and population* (PEPFAR Uganda Country Operational Plan 2015).

Relatively few documents reviewed revealed financial information relating specifically to the response to 90–90–90. In most cases costs cited were associated with the policy change for treating all PLHIV irrespective of CD4 cell count as per the WHO 2015 ART guidelines—a policy change considered critical to the attainment of 90–90–90 (herein referred to as ‘test and treat’). Thus perceptions on how the targets had influenced the HIV programme budget were obtained from informants.

The majority of participants felt the targets were largely influential over allocations within donor budgets, but less so for the national HIV programme budget. One respondent in Uganda stated: ‘You go and talk to Ministry of Finance and say with respect to 90–90–90, we have these strategies, how has this changed your budgets? The answer is zero’. (R1, Uganda). When asked why, respondents stated that reallocation would not occur as domestic budgets predominantly relate to recurrent management costs. Across both settings, no respondents were aware of funds being transferred to HIV from other areas of the health budget to respond to these targets or any other previously. Respondents from TB and malaria programmes stated that they were not aware of, or did not feel that, their disease budget had suffered as a consequence of 90–90–90. A number of respondents in Ghana were optimistic that the Government of Ghana would pledge additional funds as they previously pledged 150 million Ghana Cedis (USD 98.5m 2011 equivalent) for implementation of the 2011–16 NSP at a United Nations Special Session. Other respondents were more sceptical, stating actual government disbursement was ∼50% of what was promised: ‘Financially we have not seen the government honour its funding commitments to HIV—at least on the commodity side of HIV in the past 2 or 3 years’ (R10, Ghana). One respondent in Ghana thought there could be reallocation of central funds if Government of Ghana could not mobilize sufficient resources elsewhere to honour commitments of counterpart financing for ‘test and treat’ to PEPFAR for the 2019/20 period.

Given unlikely gains from government resources in both settings, the NACs were undertaking a variety of approaches for domestic resource mobilization to close the resource gap. The Ghana NAC were awarded a grant from the United Nations Development Program for the employment of a full-time ‘Resource Mobilization Officer’ and were continuing efforts to institutionalize HIV funding as part of private companies’ corporate social responsibility, holding annual private sector roundtable breakfast meetings to seek funds. In Uganda a national tax (2% from alcoholic drinks) was to be developed: ‘AIDS Trust Fund’. However, progress in establishing the fund was uncertain due to disagreements between MOH and Uganda NAC about whether this should be managed by MOH or independently. Ghana NAC passed legislation in 2015 for the development of a similar HIV fund in Ghana. The volume of funding obtained from these means, however, is limited and will not be sufficient to close the resource gap in either country.

Respondents in both countries felt that donor funding was very responsive to the targets set by UNAIDS. A number of respondents commented on the well-coordinated relationship of these actors at the global level—UNAIDS setting targets that are aligned with the WHO treatment guidelines and financed by PEPFAR at the national level, in particular for the rollout of ‘test and treat’. Respondents in Uganda commented not just on the volume of additional resources, but an increase in the rate of disbursement of PEPFAR and Irish Aid in response to ‘test and treat’. At the time of interviewing, both countries were mid-way through their current funding period from The Global Fund, but in Ghana where The Global Fund are the major funder, discussions had occurred with the Portfolio Manager regarding reprogramming of funds to accommodate commodities for 90–90–90 and also to frontload funds if necessary, indicating the alignment of interests of global health actors in meeting the targets:

*Global Fund has even scaled-up funding, you know, so that we will be able to achieve these targets to the extent that they are even ready to front-load some of the funds so as that we can scale-up - especially testing* (R2, Ghana).

In Ghana, PEPFAR will frontload 24 million USD for ‘test and treat’ for 2017/18 until Government of Ghana can mobilise their own resources to fund the 2019/20 period. While PEPFAR have previously contributed to the response in Ghana this has been in the realm of 14 million USD for provision of technical assistance only and not for programme support or commodities.

### (B) What was the degree of transfer?

Direct transfer of a target or programmes from one jurisdiction to another is referred to as ‘copying’ by Dolowitz and Marsh. Throughout most documents, both countries appear to have directly adopted the 90–90–90 targets without adaptation; in reality, slightly lower or adapted targets may have been transferred. For example, in Ghana, 90–90–90 was originally only intended for key populations and not the general population (Table 4). Further, whilst there are statements of adopting 90–90–90 there is clear intention to put policy, programme and financial support for viral load on a lower priority, however, until further into the second half of the time period for 90–90–90 given the expense of viral load machines and current low levels of access (coverage <10%) to viral load testing (Table 5). One respondent commented:

*even though we have started now we are actually starting slowly, because of the available commodities* (R6, Ghana).

In Uganda, the NSP (2015/16–2019/20) and PEPFAR Country Operational Plan state target ART coverage levels of 80% of PLHIV rather than then 81% that is cited in the UNAIDS 90–90–90 targets. Uganda’s marginally lower target of may be described as a ‘combined response’, which pertains to a mixture of different policies relevant to the target as the 80% may instead reflect the adoption of ‘universal access’ targets (World Health Organization 2013).

### (C) What are the constraints on transfer?

The most commonly cited constraint on transfer by informants across both settings is the inadequate financial and human resources for the response. Limitations on commodities were frequently cited: HIV test kits, ART for treating all PLHIV and viral load testing. Both countries identified a large resource gap for the 5-year period of their NSPs, which is intended to include the response to 90–90–90: 105.42 million USD in Ghana NSP and 918.40 million USD in Uganda (Ghana AIDS Commission 2016; Uganda AIDS Commission 2016). Despite efforts to mobilize, and better utilize resources for the response, respondents from both settings expressed concern that the resource gap would not be closed, with one respondent in Ghana stating:

*The truth and the reality is that if we are not careful then we will be affected by this funding gap. Those who are putting money forward say the money is coming, but money is not coming and so can we find a way to slow down. It means that we are not setting ourselves to meet the target* (R6, Ghana).

Respondents in Ghana reported a higher number of alternate constraints to the transfer of the targets than in Uganda. One respondent commented on constraints observed in reallocating resources from more generalized programme scale up to selected regions, noting that initially there had been little political support for prioritized programmes over universal coverage. Further to this, the respondent noted that limitations in epidemiological information at lower administrative units added a fair amount of uncertainty in programme planning and resource allocation for the response. Conversely, four respondents felt very confident in the location of PLHIV for informing programmes at local levels. Respondents with positions relating to implementation discussed concerns over health system capacity in regard to the uptake of newly developed task-shifting guidelines (not released at time of publication). Whilst these factors had not influenced the adoption of the targets, they are likely to influence the policy-practice gap. One respondent phrased this succinctly:

*So it feels a bit like 90*–*90*–*90 has been launched in name and targets have been put in place and there is a roadmap, very high level, but there is no detailed planning as yet and it is not clear yet what the output will be* (R17, Ghana).

### (D–F) Who was involved in transfer, from where were lessons drawn, and why did transfer occur?

#### Findings supplemented by the GHAPP framework

Despite the notable constraints on transfer of 90–90–90 that persist in both settings, evidence exists of a response to the 90–90–90 targets, with substantial programmatic shifts, supported by donor funding and domestic resource mobilization. The primary reason, or a comprehensive list of all causative factors, influencing transfer of the targets, cannot be fully known, however, we identify potential influences utilizing the 10 factors described in the GHAPP framework (Box 2).

The network of actors involved in the transfer of the target was diverse but broadly similar across Ghana and Uganda (Table 3); comprised of actors at global and local levels from a variety of sectors within and outside health (Factor 3—composition of actor networks). In Uganda, however, PEPFAR has a much more dominant role, funding over 70% of the HIV programme. There was no major opposition to the transfer of the targets noted by respondents from either setting; however, a senior implementer in Ghana voiced they had wanted adoption of more modest and realistic targets given perceived implementation challenges and resource gaps: ‘I kept mentioning it is not possible and we won’t do it until we get the money – people were feeling that I was going to be a stumbling block’ (R6, Ghana) (Factor 5—allies and opponents). UNAIDS Geneva, utilizing its widely accepted role as a technical leader in the HIV response, created awareness of the targets through several participatory venues for discussion with policy-makers from sub-Saharan Africa, including Ghana and Uganda: launch at International AIDS Society meeting in Melbourne, Australia (2014), United Nations retreat in Addis Ababa (2015) and formal adoption by the African Union at a meeting in Johannesburg in 2015 (Factor 1—leadership). Once passed down to the national level, the local health networks in both settings, was described as well governed by all respondents (particularly in comparison to the ‘disorganized past’), with all actors participating in the network clear on their roles and responsibility in the response, with NACs coordinating the effort (Factor 2—governance). In Uganda, there was a clear role of civil society and affected populations who had bought into the targets and were advocating for a response (Factor 10—role of affected groups). Consequently, the national launch for 90–90–90, organized by UNAIDS and Uganda NAC, was held at the main local organization for people living with HIV, which was cited by many as playing an important role in acceptance of the targets locally, in particular by affected groups. In Ghana, the Director General of the NAC was named by many as a clear leader in the response, acting as a policy entrepreneur, as a result of her position on central UNAIDS boards (Factor 1—leadership), which may explain the vocal response to 90–90–90 (Table 4) despite the significant financial and health system constraints demonstrated in the USAID reports: ‘The Director General is on the panel of the UNAIDS and so she would really want to demonstrate clearly our commitment [to 90–90–90]’. R1, Ghana. In Uganda, such a significant portion of the programme is funded by PEPFAR, who appear to have been able to relatively quickly refocus their programme activities from general nationwide scale up to district programming (Factor 6 – funding). In both settings, it appears that lessons were also drawn from mathematical modelling analyses, typically conducted and funded by US Government agencies; however, the extent to which these studies influenced actual programme activities is unclear (Factor 3 – composition of actor networks). In Uganda, a respondent explained that utilizing modelling, a number of programme scenarios for the HIV response were simulated, including one to meet 90–90–90 and another scenario (‘feasible maximum’) that estimated the programme coverage achievable with the resources realistically to be mobilized in the country. The latter scenario was selected and incorporated into the NSP, perhaps explaining the slightly lower targets adopted.

The health networks in both settings appear to have developed highly effective framing strategies for encouraging a response to the 90–90–90 targets (Factor 4 – framing strategies). Respondents from both countries demonstrated recognition of the benefits of meeting the policy target, both clinically and at the population level. Specifically, interviewees spoke of the merits of early treatment, the deployment of effective interventions (Factor 9—tractability), and the importance of eliminating the AIDS epidemic (the ‘policy goal’). The lower severity of the epidemic in Ghana could have been prohibitive in ensuring rapid transfer of the 90–90–90 targets (Factor 5 – severity). However, the network of actors appear to have still been effective in facilitating marked policy and programme change by utilizing framing strategies (Factor 4) – that the low HIV prevalence in Ghana should not encourage complacency, but should instead motivate desire to eradicate HIV so that the country may demonstrate leadership in West Africa, with one stating: ‘only right to be able to address this issue and eliminate HIV from this country while the epidemic is still small’ (R11). Such phrasing of ‘only right’ also demonstrates the now well-embedded norms of human rights within the HIV response that encourage a response, such as the right to health, and thus access to efficacious medicines, which is aligned with the 90–90–90 treatment targets (Factor 7—norms).

Whilst all respondents voiced that transfer was voluntary or ‘not an imposition’, transfer of the target was clearly attributed to the mandate to respond to the United Nations (Table 3). Some respondents described a feeling of pressure or competition within the international community, put by one Ugandan respondent (Factor 7—norms): ‘that you just look terrible if you do not pick up the target’(R9). The majority of informants across both settings said that availability of funding was not a reason to adopt the 90–90–90 targets given the resources available would be insufficient for the complete response. However, it did still appear that there was a strong financial motivation for accepting the targets (Factor 6—funding). PEPFAR have largely driven the response to ‘test and treat’ in both settings, providing funding for commodities; funding for commodities was made available further to necessary revisions in clinical treatment guidelines. One respondent in Uganda succinctly highlighted the requirement to respond to the United Nations and for securing funding:

*The 90*–*90*–*90 targets are global targets and that is guidance that cascades from UNAIDS and WHO - member countries have to respond to that, but also that is essentially an addendum to Sustainable Development Goals and a framework for funding. If you are not there then you cannot get funding because this is the framework* (R6, Uganda).

It is possible the environment of declining donor funding contributes to greater desire for national level decision-makers to adopt targets in efforts to compete for more funding. The policy goal of eliminating the AIDS epidemic is used not only as an advocacy message to secure financing from current donors, but also to seek additional funding from new donors, private companies and through novel financing mechanisms created through the MOH, such as the AIDS Trust Fund. This was particularly exemplified in Ghana where there are requirements for counterpart financing. A Ghanaian respondent described how the commitments to 90–90–90 in the NSP was intended for advocacy for resource mobilization:

*You always have to make a strong recommendation with a strategic document such as this [the NSP]… We all need to make our case for resources* (R3, Ghana).

All respondents in both countries continue to be in favour of ambitious health targets to help galvanise action and leadership for issues of public health significance. A small number of respondents in both countries did view the targets more as ‘just a slogan’ or symbolic. Many felt recognition for progress should be just as important as meeting targets and there should be better follow up and evaluation by UN agencies to help countries that fall behind.

## Discussion

The setting of health targets by global health institutions intended for adoption at the national-level is increasingly commonplace. Review of the influence of these targets on policy, programme, and resources is of value to those that set the targets and those that are intended to respond. First, as global health initiatives can ascertain whether the targets they set result in any real changes to programmes, and learn from factors that facilitate and hinder adoption. Second, as it is not necessarily clear that national level decision-makers should respond to all targets set down upon them because of resource constraints on the health budget. In this study, we explore the transfer of the 90–90–90 treatment targets set by UNAIDS near to midway through the timeframe to 2020, in two countries, Ghana and Uganda, that have markedly different HIV disease burden.

Within a relatively short time frame, the policy targets were incorporated into relevant donor and national strategies, policies and operational plans in both countries to varying degrees. Concerns have been raised previously about the slow pace of policy translation in many sub-Saharan African countries, whereas we demonstrate evidence of effective and diverse health networks in both settings that utilize modified framing strategies to encourage relatively fast transfer of these targets to their national setting (Tumwesigye *et al.* 2013). However, given the timeframe for 90–90–90 is very short (5 years), it could be argued that 2 years is too slow for updating national strategies and policy guidelines. Experience from the response to the Millennium Development Goals found efforts to mobilize domestic resources in order to meet the targets was insufficient across many countries (Ooms *et al.* 2010). In this analysis we find evidence of concerted efforts to mobilise domestic revenue and to increase efficiency in the programme response. Estimates for resource needs for the response in both countries, however, indicate that despite these efforts, significant resource gaps remain (Ghana AIDS Commission 2016; Uganda AIDS Commission 2016). In Ghana and Uganda, it is not apparent that MOH budgets for the HIV programme have changed as a consequence of the targets or that resources have been reallocated from other disease areas specifically to meet these targets. While more investigation is needed, this finding could alleviate concerns that targets displace funding from other parts of the health budget. Instead, we indicate that these targets have resulted in a renewed interest and focus on domestic resource mobilization mechanisms. The adoption of novel domestic resource mobilization strategies is likely influenced by a number of factors and not wholly attributable to the 90–90–90 targets, such as the Abuja Declaration, which both countries studied still fall short of. Whether other sub-Saharan African countries exhibit similar responses is yet to be seen.

UNAIDS reported global progress to 90–90–90 at the mid-way point to 2020 (UNAIDS 2017). While Botswana has already met the targets, for most other countries in sub-Saharan Africa, there is a substantial way to go. Whilst Ghana and Uganda demonstrate their intentions to respond to 90–90 and ‘test and treat’, what will get translated into practice on the ground is likely to suffer gaps between intentions and actions. For the shift from nationwide scale up to an approach that targets populations and geographies to have the desired impact, there is requirement for a high degree of certitude in where to target limited resources or for flexibility in reallocation of resources should the evidence, upon which allocation decisions have been made, change. PEPFAR are thought to have a relatively high degree of flexibility in allocation of resources, compared with domestic resources, as evidenced by their reconfiguration in focus districts between Country Operational Plans 2015 and 2016 in Ghana and Uganda further to latest data on local epidemiology or service use, respectively (PEPFAR Ghana 2015, 2016; PEPFAR Uganda 2015, 2016). Uganda is now thought to be on track to meet 90–90–90, perhaps as a substantial portion of the HIV programme is funded by PEPFAR who with a degree of autonomy can readily reallocate resources to areas of need or greatest impact.

We find the Dolowitz and Marsh framework valuable for enabling consideration of differences in the response in two settings, but limited in explanatory power for the differences between settings, in particular, in regard to the role of actor networks. We find supplementing the Dolowitz and Marsh framework with the GHAPP useful in considering a range of factors that influenced the effectiveness of transfer. Through application of the GHAPP we see how lessons are being drawn from mathematical modelling analyses with US government agencies funding the provision of such technical assistance. We consider there to be a growing epistemic community of mathematical modellers arising in the HIV response; modellers provided the framework through which to consider how to eliminate the AIDS epidemic (that is the 90–90–90 targets) and offer a framework for considering how best to respond, thus driving national application of such methods. Mathematical modelling studies that demonstrate the cost effectiveness of ART scale up for test and treat have been criticized for being ‘theoretically optimal, but practically infeasible’ (Mikkelsen *et al.* 2017). In order for modelling to rationally inform national programme planning and policy, analyses that incorporate health system constraints are needed. Whether countries that directly apply mathematical modelling to inform HIV programme planning achieve greater impact than countries that do not, may warrant investigation.

In this study, transfer appears to be based to a large extent on the desire for international acceptance, to conform to social norms, and for receipt of funding for the HIV programme. In the policy transfer literature, Dolowitz and Marsh state their belief that transfer based upon financial necessity or for international acceptance is more likely coerced rather than voluntary, and thus may be more likely to result in policy failure due to inappropriate transfer of the policy to that context (Dolowitz and Marsh 2000). We argue, however, that coerced transfer is not always inappropriate; the 90–90–90 targets and associated policy content are based on research evidence and we do not find clear evidence that the targets have diverted resources away from other important health issues (whether such targets are still detrimental to attainment to goals of universal health coverage remains unknown). However, it is clear that context specific investigations need to be conducted and supported so that decision-makers at the national level can ascertain how beneficial a response is in terms of population health.

Follow-up with these countries would be advised to assess whether domestic resource mobilization mechanisms are established successfully and finances realized in the timeframe necessary to support 90–90–90. If funds are not realized it would be important to ascertain which aspects of the HIV programme are not funded or whether funds are later reallocated from other health issues in order to meet these targets. Further, as it is apparent large gains are intended from programme efficiencies, such as shifts to differentiated models of care, facility level implementation studies would be of value.

### Limitations

In both countries, and across the African continent, reporting of financial information for the HIV response has been limited and inconsistent. Uganda’s last National AIDS Spending Assessment covers up to 2010 only. Thus efforts to track funding allocations are weak. Analysis of budgetary changes and health outcomes would likely yield more information on the actual consequences of such targets, but will not be feasible until tracking of expenditure is institutionalized and routine. We therefore make a call for better reporting on such information in order to improve decision-making in the longer term. It was not possible to interview all desired individuals, in particular the Ministry of Finance, which would have provided insight on internal allocation processes. When interviewing senior representatives of organizations, as we did in our study, it can be difficult to obtain an honest personal opinion rather than the ‘party line’. We consider the respondents to have been relatively truthful, however, given that concerns were raised over realization of resources and as statements were largely corroborated with the document review.

## Conclusions

The 90–90–90 targets have been transferred to the national programme in Ghana and Uganda with evidence of a shift to more targeted programming and reinvigoration of domestic resource mobilization efforts. Differences in adoption of the targets between the countries studied could be attributed to variation in the composition of the actor networks involved in the transfer of the targets and the technical and financial resources available to them. Whether the targets achieve significant traction in implementation and ultimately the intended policy goal warrants follow-up. Improvements in routine collection and reporting of resource allocation and budgeting for the HIV response are recommended in order to support quantitative assessments of the influence of disease-specific targets on national HIV programme activities and across health more broadly.
